# Gene Expression Profiling Reveals Potential Players of Left-Right Asymmetry in Female Chicken Gonads

**DOI:** 10.3390/ijms18061299

**Published:** 2017-06-20

**Authors:** Zhiyi Wan, Yanan Lu, Lei Rui, Xiaoxue Yu, Fang Yang, Chengfang Tu, Zandong Li

**Affiliations:** 1 State key Laboratory for Agrobiotechnology, College of Biological Sciences, China Agricultural University, Beijing 100193, China; gxmwzy@126.com (Z.W.); nan0914@126.com (Y.L.); ruilei@cau.edu.cn (L.R.); yuxiaoxue1990@163.com (X.Y.); 2 College of Life Sciences, Peking University, Beijing 100871, China; jessamine02@163.com; 3 Annoroad Gene Technology Co., Ltd., Beijing 100176, China; m15910560696@163.com

**Keywords:** left-right asymmetry, gonads, female chicken, RNA-seq

## Abstract

Most female birds develop only a left ovary, whereas males develop bilateral testes. The mechanism underlying this process is still not completely understood. Here, we provide a comprehensive transcriptional analysis of female chicken gonads and identify novel candidate side-biased genes. RNA-Seq analysis was carried out on total RNA harvested from the left and right gonads on embryonic day 6 (E6), E12, and post-hatching day 1 (D1). By comparing the gene expression profiles between the left and right gonads, 347 differentially expressed genes (DEGs) were obtained on E6, 3730 were obtained on E12, and 2787 were obtained on D1. Side-specific genes were primarily derived from the autosome rather than the sex chromosome. Gene ontology and pathway analysis showed that the DEGs were most enriched in the Piwi-interactiing RNA (piRNA) metabolic process, germ plasm, chromatoid body, P granule, neuroactive ligand-receptor interaction, microbial metabolism in diverse environments, and methane metabolism. A total of 111 DEGs, five gene ontology (GO) terms, and three pathways were significantly different between the left and right gonads among all the development stages. We also present the gene number and the percentage within eight development-dependent expression patterns of DEGs in the left and right gonads of female chicken.

## 1. Introduction

Although mammals have apparently symmetric gonads, most avian species including ducks [1] and chickens [2] exhibit an unusual asymmetry in gonadal development. In female birds (genetically ZW), only the left gonad becomes a functional ovary, whereas the right ovary completes regression at the adult stage. In male birds (genetically ZZ), testicular development occurs bilaterally [3,4].

In chick embryos, gonadogenesis begins approximately on day 3 of development during Hamburger Hamilton (HH) stage 23 (E3) [5]. At this stage (the “indifferent stage”), there is no detectable morphological asymmetry between the left and right embryonic gonads of either sex. However, a greater proportion of the circulating primordial germ cells (PGCs) colonize the left than the right gonad [6,7]. After sexual differentiation, the morphological appearance of the gonads is initially similar in males and females (HH28 E5.5), but by HH36 (E10.5), the gonads in males and females are distinctly different. The morphological differences between the left and right gonads in the female become more pronounced, while the asymmetry between male gonads diminishes. By HH44 (E18.5), only a few germ cells are randomly found throughout the core region in the right ovary, whereas the germ cells are distributed predominately in the cortex in the left ovary [8].

In addition to the morphological asymmetry between the left and right gonads, several genes are related to the asymmetrical development and the right ovary degeneration [9,10,11]. For example, *Bmp7* is expressed asymmetrically in the indifferent ridges of both sexes [10], whereas the estrogen receptor (ER) is expressed in the left but not the right cortex of both sexes [12]. *Pitx2* has been shown to control asymmetric gonadal development in both sexes of the chick and can rescue the degenerative fate of the right ovary [11].

The aforementioned studies focused on early embryonic events mainly based on histological descriptions or on mRNA expression profiles of a distinct gene subset. However, the comprehensive molecular descriptions of this differential expression are far from being completely understood. Many questions have still been left unanswered; for example, is there a global gene expressional regression in the development of the right ovary along with the morphological regression process? What are the molecular factors underlying side-specific development in female chicken gonads? With the advent of next-generation sequencing (NGS), RNA-seq has been used to assess chicken gonadal sex- and side-biased genes at different developmental stages [13,14,15].

Here, we provide a comprehensive description of the side-specific differential sexual development of female chicken gonads at the level of gene expression on E6 (the onset of morphological differentiation), E12, and post-hatching day 1 (D1, the end of embryonic development), and we discuss the molecular basis of this unusual asymmetry. There was no global regression in the development of right gonad from E6 to D1. Several new genes, gene ontology (GO) terms, and pathways were identified as important for the left-right asymmetry of the gonad. A total of 111 genes, five GO terms, and three pathways were significantly differences between the left and right gonads among all the development stages. We also present the number and the percentage of genes within eight development-dependent expression patterns of differently expressed genes (DEGs) in the left and right gonads of female chicken.

## 2. Results

### 2.1. General Analysis of Gene Expression Profiles among Different Samples

The female chicken gonad gene expression profiles on E6, E12, and D1 were investigated using RNA-Seq. After data filtering, a total of 205.0 M 50 bp clean reads were acquired, ranging from 16.6 to 18.0 M reads per sample. Of the clean reads, more than 86% were mapped to the chicken genome (galGal4) for each sample. More than 82% of these clean reads uniquely mapped to specific regions of chicken genome (Table 1). Only the uniquely mapped reads were used for further analyses. The average percentage of the uniquely mapped reads of exonic regions was 82.5%, indicating a high level of coverage of the actual transcribed sequences (Table S1).

**Figure 1:**
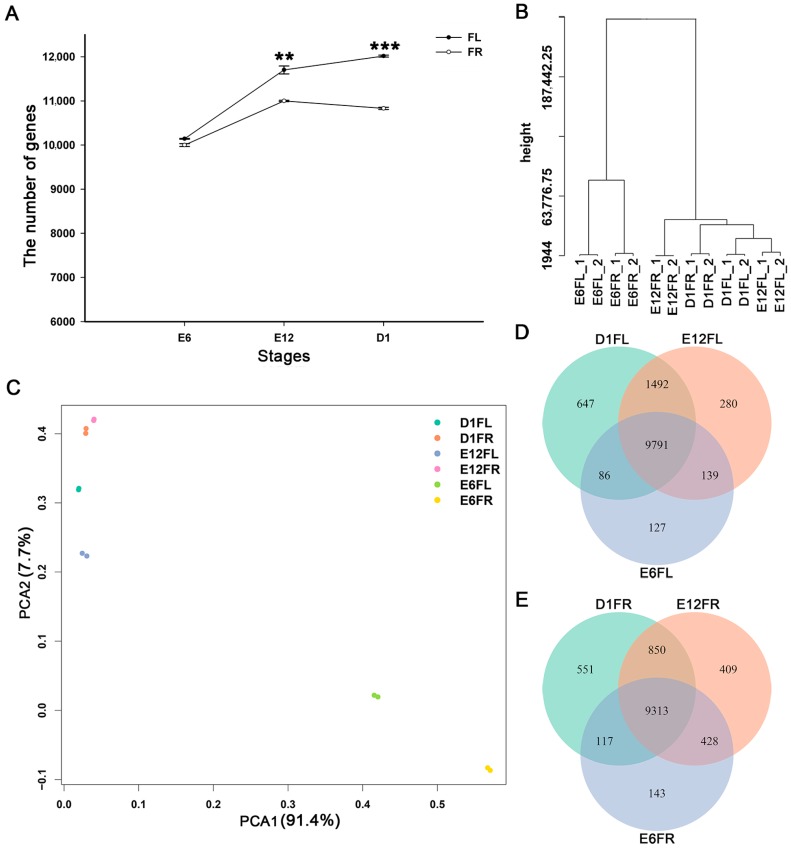
Analysis of global gene expression among different samples. (**A**) Number of genes expressed in the left and right gonad. Statistically significant differences in the number of genes in the left and right gonad were analyzed by Student’s *t*-test; ** *p* < 0.01, *** *p* < 0.001 (L: Left, R: Right); (**B**) Hierarchical clustering of all the biological samples; (**C**) The principal component analysis (PCA) of the RNA-seq data for all the biological samples. Venn diagrams indicate the overlap of global gene expression signatures identified at stages E6, E12, and D1 of the female left gonad (**D**) and right gonad (**E**). E6FL Female left gonad on E6, E12FL Female left gonad on E12, D1FL Female left gonad on D1, E6FR Female right gonad on E6, E12FR Female right gonad on E12, and D1FR Female right gonad on D1.

All genes (≥1 RPKM (reads per kilobase million mapped reads)) from the 12 samples are listed in Table S2. There were 10,143 ± 9.9, 11,702 ± 89.1, 12,016 ± 23.3, 10,001 ± 32.5, 11,000 ± 14.8, and 10,831 ± 25.5 genes expressed in E6FL (female left gonad on E6), E12FL (female left gonad on E12), D1FL (female left gonad on D1), E6FR (female right gonad on E6), E12FR (female right gonad on E12), and D1FR (female right gonad on D1), respectively. The number of genes detected in right and left gonads at E6 was almost equivalent; however, the number of genes detected in the right gonad was significantly lower than that in the left gonad at E12 and D1 (Figure 1A).

A hierarchical clustering analysis and principal component analysis (PCA) were performed to gain insight into the relationships among different samples (Figure 1B,C). The cluster analysis generated two large groups corresponding to “E6” and “E12 to D1”, regardless of the gonad side. However, in the “E12 to D1” group, E12FL was clustered together with D1FL rather than E12RL, suggesting that the difference between the left and right is greater than that between E12 and D1. Data from E12FR and D1FR were more similar with the gonad on E6; this indicated that the differentiation process of right gonad was inhibited (Figure 1B). Almost all the biological duplicates were also clustered together, showing the efficiency of the method and confirming the relevance of the samples. The PCA result was consistent with that of cluster analysis (Figure 1C). The first component (91.4% variance explained) separated samples based on development stage.

The distribution of differential expression genes among the gonads was revealed using a Venn diagram (Figure 1D). In the female left gonads and right gonads, 9791 and 9313 genes were expressed in all three developmental stages, respectively.

### 2.2. Right-Left Expression Profile

**Figure 2:**
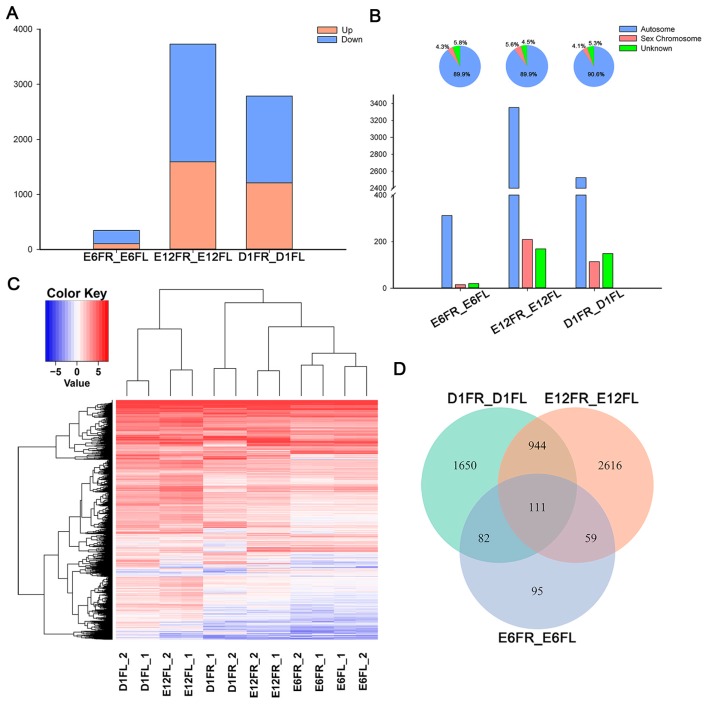
Analysis of differently expressed genes (DEGs) between the left and right gonads. (**A**) Number of DEGs between the left and right gonads; (**B**) Chromosomal allocation of DEGs in the left and right gonads; (**C**) Hierarchical clustering of DEGs in all of the biological samples. Each row represents a gene and each column represents a sample. Each cell in the matrix corresponds to an expression level, with blue for under-expression, red for overexpression, white for gene expression close to the median (see color scale). (**D**) Venn diagrams show the shared and unique DEGs obtained from each pairwise comparison between the left and right gonads. E6FR_E6FL, E6FR vs. E6FL; E12FR_E12FL, E12FR vs. E12FL; D1FR_D1FL, D1FR vs. D1FL.

To identify the side-specific genes of female gonads, we compared the gene expression profiles between the left and right gonads at E6, E12, and D1. A total of 347 DEGs (104 upregulated and 243 downregulated) were found in E6FR compared with E6FL. The number of DEGs in E12FR compared with E12FL (3730, 1591 upregulated and 2139 downregulated) was higher than in D1FR compared to D1FL (2787, 1209 upregulated and 1578 downregulated) (Figure 2A, Table S3). A ten-fold increase in the left-right asymmetry gene number was observed between E6 and E12, the period the gonads differentiate. From E12 to D1, the morphological differences between left and right gonad increased, whereas the number of DEGs between the right and left gonad was decreased.

Chromosomal allocation of DEGs was analyzed based on the annotated gene data (Figure 2B). DEGs between E6FR and E6FL were annotated to the autosome (312, 89.9%), the sex chromosome (15, 4.3%), or to the unknown chromosome (20, 5.8%). DEGs between E12FR and E12FL were located on the autosome (3352, 89.9%), the sex chromosome (209, 5.6%), or on the unknown chromosome (169, 4.5%). DEGs between D1FR and D1FL were found on the autosome (2524, 90.6%), the sex chromosome (114, 4.1%), or on the unknown chromosome (149, 5.3%).

All DEGs between the left and right gonads in different development stages were classified using hierarchical clustering (Figure 2C). A total of 5557 DEGs were clustered into four main groups according to their expression pattern similarity. The clustered groups were not fully consistent with our previous observations, and E12FL remained clustered together with D1FL rather than E12RL.

When combined, there were 111 DEGs among all the development stages (Figure 2D). A list of these 111 DEGs are shown in Table S4. In the 111 DEGs, six genes (5.4%) were located on the sex chromosome, and interestingly, all of them were on the Z chromosome. The results of RNA-seq showed that *PITX2* was asymmetrically expressed in the gonads on E6 and D1, whereas *PITX3* was expressed asymmetrically on E12 and D1.

**Figure 3:**
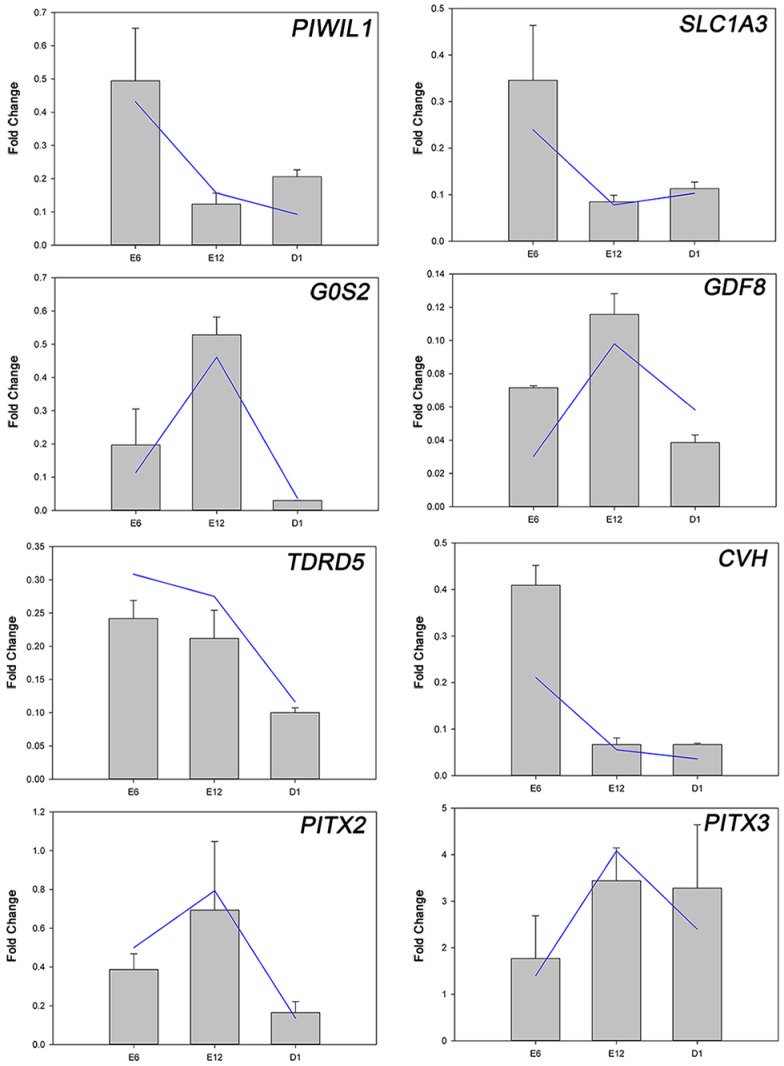
Expression patterns of eight DEGs involved in left-right asymmetry by RNA-seq (blue line) and qRT-PCR (gray bar). The quantitative real-time RT-PCR (qRT-PCR) values were normalized relative to the expression levels of *GAPDH* in the same cDNA sample. Expression data are presented as the expression values of genes in the right gonads relative to that in the left sample. Data are expressed as the mean ± SD of two biological replicates.

*PITX2*, *PITX3*, and six of the 111 DEGs (*PIWIL1*, *SLC1A3*, *TDRD5*, *CVH*, *G0S2*, and *GDF8*) were further selected to validate the gene expression levels by quantitative real-time RT-PCR (qRT-PCR) (Figure 3). These qRT-PCR results were consistent with those obtained by RNA-seq. The expression levels of *PIWIL1*, *SDF*, *TUDOR*, *CVH*, *G0S2*, and *GDF8* in the right gonad were significantly lower than the expression levels in the left gonad among all the development stages.

Except for *PIWIL1*, *CVH*, *TDRD5*, and *PITX2*, other genes related to germ cells including *TDRD1*, *TDRD2*, *TDRD6*, *TDRD8*, *TDRD9*, *GRIP2*, *DND1*, *DAZL*, and *MAELSTROM* were asymmetrically expressed among the three development stages (Table S4).

To understand the functions of these DEGs, GO and Kyoto Encyclopedia of Genes and Genomes (KEGG) analyses were performed. Summaries of the classifications of the DEGs into the three GO sub-ontologies (Biological process (BP), Cellular component (CC), and Molecular function (MF)) are presented in Figure S1. Further enrichment analysis was performed, and the significantly regulated GO terms are listed in Table 2 and Table S5. Between E6FR and E6FL, the significantly regulated BP categories in DEGs were Piwi-interactiing RNA (piRNA) metabolic process and DNA methylation involved in gamete generation. The top three CCs were pole plasm, germ plasm, and P granule. There were no significantly affected GO terms in MF categories (Table 2). The common significantly enriched GO terms of DEGs among all stages were related to piRNA metabolic process, germ plasm, chromatoid body, P granule, pi-body, and pole plasm (Table S5).

**Figure 4:**
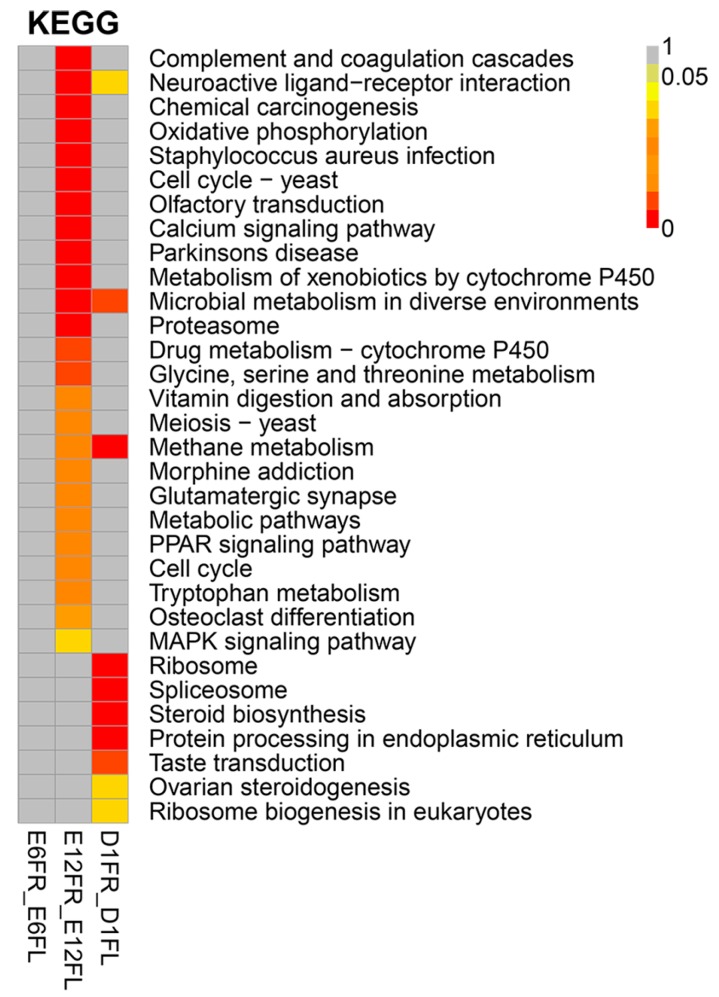
Heatmap of Kyoto Encyclopedia of Genes and Genomes (KEGG) annotations for DEGs between the left and right gonads. Each row represents a metabolic pathway. Different colors represent the *q*-value. E6FR_E6FL, E6FR vs. E6FL; E12FR_E12FL, E12FR vs. E12FL; D1FR_D1FL, D1FR vs. D1FL.

For pathways analyses, we mapped the DEGs to KEGG orthologs and performed an enrichment analysis with the whole transcriptome as the background. In E6, there were no significantly enriched pathways between the left and right gonads. In E12FR and E12FL, 493 DEGs were significantly enriched in 25 KEGG pathways. In D1FR and D1FL, 141 DEGs were significantly enriched in 10 KEGG pathways. The three common DEG pathways between E12 and D1 were related to neuroactive ligand-receptor interaction, microbial metabolism in diverse environments, and methane metabolism (Figure 4). Figure 4 also shows the significantly enriched pathways between the left and right gonads.

**Figure 5:**
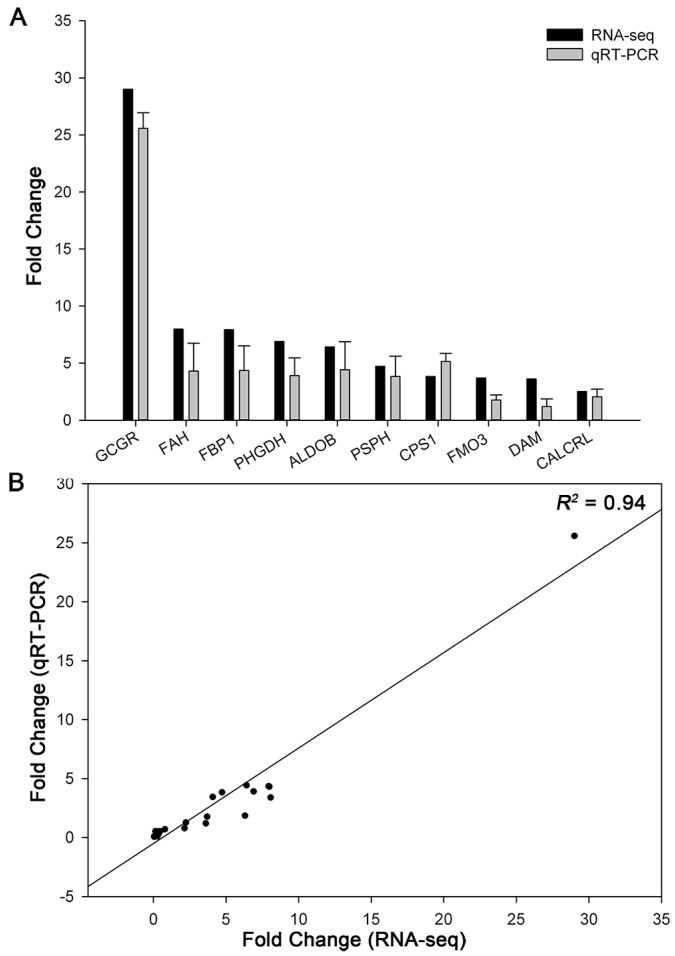
Verification of differently expressed genes by qRT-PCR. (**A**) The expression of 10 genes was validated using qRT-PCR and compared with the expression levels obtained from RNA-seq. The qRT-PCR values were normalized relative to the expression levels of GAPDH in the same cDNA sample. Expression data are presented as expression values of genes in right gonads relative to that in left sample. Data are expressed as the mean ± SD of two biological replicates. (**B**) Correlation of 29 gene expression results obtained from RNA-seq and qRT-PCR.

Based on the DEGs pathway enrichment results, a qRT-PCR of 21 DEGs related to neuroactive ligand-receptor interaction, microbial metabolism in diverse environments, and methane metabolism was performed to validate the differential gene expression obtained by RNA-seq. The expression levels of 10 genes in these 21 DEGs are shown in Figure 5A. Using the results shown in Figure 3 and Figure 5A, we calculated the correlation efficiency of RNA-seq and qRT-PCR data and observed a strong correlation between the two (*R*^2^ = 0.94) (Figure 5B). These results further indicated that the gene expression patterns as obtained by qRT-PCR corroborated those obtained by RNA-seq (Figure 5).

### 2.3. Development-Dependent Gene Expression Patterns

**Figure 6:**
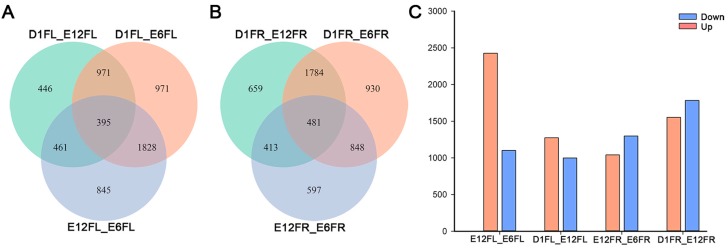
Differential gene expression in the pairwise comparison of developmental stages. Venn diagrams indicate overlap of all DEGs obtained from each pairwise comparison between developmental stages in the left gonad (**A**) and right gonad (**B**). (**C**) Number of DEGs between developmental stages in the female gonads. E12FL_E6FL, E12FL vs. E6FL; D1FL_E12FL, D1FL vs. E12FL; D1FL_E6FL, D1FL vs. E6FL; E12FR_E6FR, E12FR vs. E6FR; D1FR_E12FR, D1FR vs. E12FR; D1FR_E6FR, D1FR vs. E6FR.

The differential gene expression analysis results in the pairwise comparison between developmental stages are shown in Figure 6. With respect to the female left gonads, a total of 5917 genes were found to be differentially expressed during development: 3529 between stages E6FL and E12FL, 2273 between stages E12FL and D1FL, and 4165 between stages E6FL and D1FL (Figure 6A). For the right gonads, a total of 5712 DEGs were identified: 2339 between stages E6FR and E12FR, 3337 between stages E12FR and D1FR, and 4343 between stages E6FR and D1FR (Figure 6B). Interestingly, the number of DEGs in the right gonad was increased along with regression; however, the number of DEGs in the left gonad DEGs, especially the number of upregulated genes, decreased with differentiation (Figure 6C).

**Figure 7:**
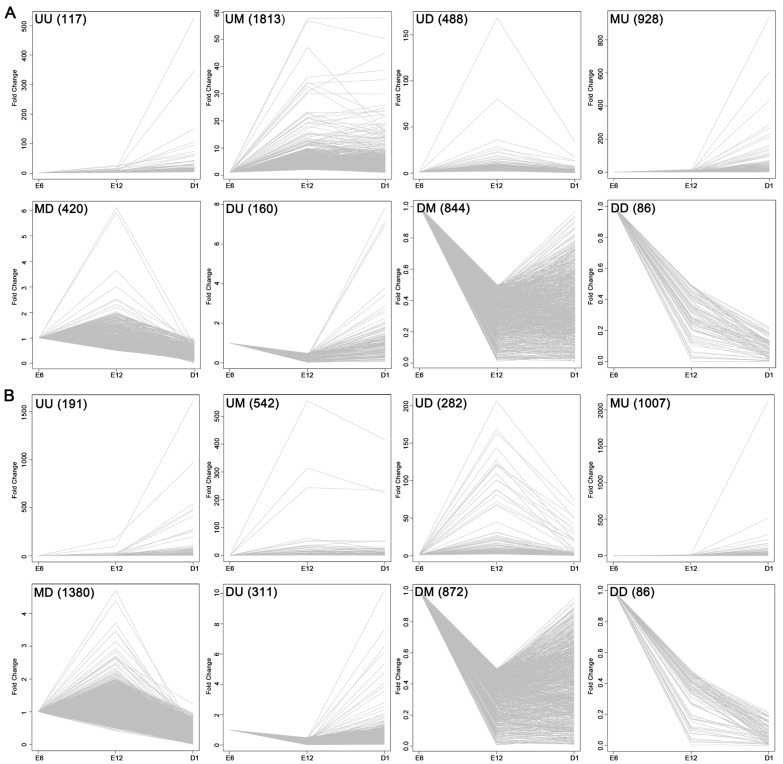
Development-dependent patterns of DEGs in the left gonad (**A**) and the right gonad (**B**). Data were obtained from the union of the DEGs between sequential developmental stages, with the younger developmental stage used as the denominator. Genes were grouped into Up (U; “upregulated” based on fold change (FC) >2 and false discovery rate (FDR) <0.05), Down (D; ‘downregulated’ based on FC > 0.5 and *p*-value < 0.05), or Maintain (M; “no change” based on 0.5 < FC < 2 or *p*-value > 0.05). The number shown in each box indicates the number of genes within the pattern.

**Figure 8:**
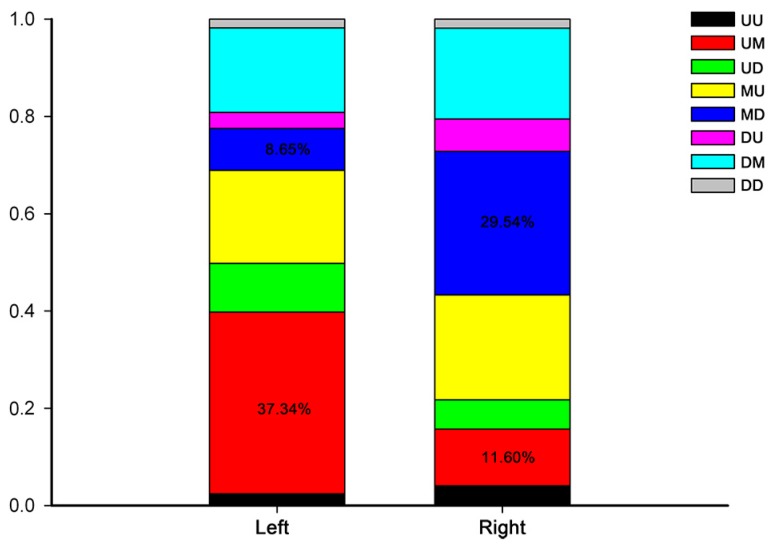
The percentage of genes within each expression pattern in the left and right gonads. The eight possible patterns included up-up (UU), up-maintain (UM), up-down (UD), maintain-up (MU), maintain-down (MD), down-up (DU), down-maintain (DM), and down-down (DD).

Finally, we performed a time course differential gene expression analysis by comparing the expression pattern of all DEGs over the three developmental stages. This approach revealed eight development-dependent patterns of DEGs. The overall development-dependent patterns are shown in Figure 7 and Figure 8, and Tables S6 and S7. Genes were non-randomly represented across all patterns. The number of genes that significantly continuously changed (UU, UD, DU, DD; U: upregulated; D: downregulated) was 851 and 870 in the left and right gonads, respectively. By comparing the ratios of different patterns between the left and right gonads we found that the group up-maintain (UM) accounted for approximately 37.34% of the DEGs in the left gonad, but only 11.60% in the right gonad. However, the MD group accounted for about only 8.65% of the DEGs in the left gonads, whereas it accounted for 29.54% of the DEGs in the right gonads (Figure 8).

## 3. Discussion

The chicken embryo represents a suitable model for studying sex determination and gonadal asymmetry. Many sex-biased genes have been identified, such as *SOX9*, *SF1*, *GATA-4*, and *Lhx9* [16,17,18]. However, only a small number of genes have been identified as expressed in a side-biased manner. Here, we provide the gene expression profiles of female chicken gonads and identify novel candidate side-biased genes by RNA-seq. In addition, a detailed view of the female chicken gonads transcriptome has been revealed by comparing three development stages.

After data filtering, 16.6 to 18.0 M reads per sample were acquired. Based on the results of saturation curves analysis, the gene quantification was reliable for genes with medium or high expression levels. However, some of rare mRNA will be missed.

As gonadal development proceeds, the morphological differences between the female left and right gonads become more pronounced [8]. However, our results showed that the number of DEGs between the left and right gonads increased from E6 to E12, and then decreased from E12 to D1. We found that the major DEGs were located on the autosome in the third development stage, which is consistent with a previous report [13]. There were no obvious differences in the percentage of DEGs annotated to the autosome in all development stages. The hierarchical clustering analysis and PCA revealed that E6FL and E6FR were clustered together, and E12FR and D1FR were more similar to the gonad at E6. These results show that there is not a global regression of gene expression profiles in the development of the right gonad.

In addition to morphological asymmetry, asymmetric germ cell distribution was also reported. Previous studies have suggested that the number of PGCs and germ cells in the left gonad was greater than that in the right gonad [7,19,20]. Accordingly, we especially focused on specific germ cell markers including VASA (CVH) [21], PIWIL1 [22], DAZL [23], and TDRD [24]. PIWIL1 plays a central role during gametogenesis by repressing transposable elements and preventing their mobilization, which mediates the repression of transposable elements during meiosis by forming complexes composed of piRNAs and Piwi proteins and governs the methylation and subsequent repression of transposons [25]. Recent studies identified the TDRD members, including TDRD1, TDRD5, TDRD7, and TDRD9, as participating in the Piwi pathway and/or retrotransposon silencing [26,27,28,29]. *TDRD* and *VASA*, encoding germ plasm components, were required for germ cell formation in many metazoan species [24]. Piwi was co-localized with *VASA* mRNA in germ cells [30]. We found that the expression levels of CVH, PIWIL1, and TDRD5 were significantly higher in the left gonad than the right gonad in female chicken according to both RNA-seq and qRT-PCR. In addition, the RNA-seq results also showed that *MAELSTROM* and the other TDRD members, including *TDRD1*, *TDRD2*, *TDRD6*, *TDRD8*, and *TDRD9*, were expressed asymmetrically among the three development stages. *MAELSTROM* encodes a protein that co-localizes with VASA and a RDE1/AGO1 homolog and plays a crucial role in the piRNA pathway [31].

Our GO analyses further revealed that the strongest differences occur in the piRNA metabolic process (Piwi-associated RNA metabolic process), pole plasm, chromatoid body, P granule, pi-body, and germ plasm. The chromatoid body, a germ-cell specific RNA-processing center, has been suggested to be the mammalian counterpart of germ plasm [32]. *VASA* mRNA co-localizes with both *Drosophila melanogaster* germ plasm and the mouse chromatoid body. Furthermore, TDRD5 is required for retrotransposon silencing, chromatoid body assembly, and spermiogenesis in mice [29]. The P granule, a small cytoplasmic, non-membranous RNA/protein complex, aggregates in the primordial germ cells of nematodes [33].

Pluripotency genes including *cPouV*, *cNanog*, *cSox2*, and *ERNI* were also found to be expressed asymmetrically in embryonic gonads [34]. Scheider et al. reported that pluripotent markers such as cNanog and cPouV did not display asymmetric expression in male or female gonads at higher development stages [13]. However, in our study, RNA-seq analysis revealed that *cPouV* and *cNanog* were expressed at higher levels in the left gonad than the right gonad on both E6 and D1. On E12, the expression levels of *cPouV* and *cNanog* between the left and right gonads were not significantly different. The expression levels of *cSox2* and *ERNI* between the left and right gonads were not different among all development stages.

The asymmetric distribution of germ cells was also related to cell proliferation. Our RNA-seq and qRT-PCR analyses have shown that *G0S2* and *PITX*2 were preferentially expressed in the left gonad among all the development stages. G0S2 is a multifaceted protein involved in proliferation, apoptosis, and metabolism [35]. PITX2 was reported to regulate gonadal cell proliferation and morphogenesis. Misexpression of *PITX2* in the right gonad is sufficient to induce symmetric development of the gonads and rescue the degeneration of the right gonad [11].

To evaluate the development-dependent transcriptomic activities in the left and right gonads, we performed a time course differential gene expression analysis by comparing any two adjacent developmental stages. Here, we present the number and percentage of genes within each expression pattern. Unexpectedly, we found that the percentage of genes within the patterns of UM and MD was markedly different between the left and right gonads. Future studies will focus on the molecular mechanism of these differences in more detail.

## 4. Materials and Methods

### 4.1. Embryo Incubation and Tissue Collection

Fertilized White Leghorn chicken eggs (*Gallus gallus*) were obtained from the Experimental Station of China Agricultural University (Beijing, China) and bred at 37.5 °C under a relative humidity of 55–65% (P-008B Biotype, Showa Furanki, Saitama, Japan). All animals received humane care as outlined in the Institutional Guidelines of the Care and Use of Laboratory Animals at China Agricultural University (Permit Number: SKLAB-2015-06-06, Beijing, China).

The embryonic gonads were collected on embryonic day 6 (E6), E12, and post-hatching day 1 (D1). At each stage, the left and right gonads were dissected separately, placed individually in TRIzol reagent (Invitrogen, Carlsbad, CA, USA) and stored at 4 °C. Embryonic blood cells (1 μL) were used for sex determination as previously described [36]. After sexing, 6–10 gonads, depending on the stage, were pooled from each replicate according to sex, stage, and side. Each set included two biological replicates. Pooling has been documented as an appropriate approach to prepare samples for expression analysis [37].

### 4.2. Library Construction and Sequencing

Total RNA was extracted using TRIzol reagent (Invitrogen, Carlsbad, CA, USA) following the manufacturer’s instructions and was treated with DNase I. RNA integrity and concentration were assessed using the RNA Nano 6000 Assay Kit of the Bioanalyzer 2100 system (Agilent Technologies, Santa Clara, CA, USA). The sequencing libraries were generated using the NEBNext^®^ Ultra™ RNA Library Prep Kit for Illumina^®^ (#E7530L, NEB, Ipswich, MA, USA) following manufacturer’s recommendations. Library concentration was measured using the Qubit dsDNA BR assay kit (Life technologies, Waltham, MA, USA). Insert size was assessed using the Agilent Bioanalyzer 2100 system (Agilent Technologies, Santa Clara, CA, USA), and qualified insert size was accurately quantified using StepOnePlus™ Real-Time PCR System (Thermo Fisher Scientific, Waltham, MA, USA). The libraries were sequenced by Annoroad (Beijing, China) on an Illumina NextSeq 500 platform (Illumina, Santiago, CA, USA), and 50 bp single-read reads were generated. The RNA-seq data from the 12 samples have been submitted to the National Center for Biotechnology Information Sequence Read Archive with accession number SRP081829.

### 4.3. Data Filtering and Alignment

Raw reads generated by the Illumina NextSeq 500 were filtered to remove low quality reads (reads containing more than 15 % bases with a *q*-value ≤ 19), adaptor-containing reads, and reads containing more than 5% ambiguous nucleotides. After pre-processing, clean reads were obtained and aligned to the chicken genome (galGal4) using TopHat v2.0.12 (Baltimore, MD, USA) [38]. Reads mapped to multiple locations and unmapped reads were excluded from gene expression analysis.

### 4.4. Gene Expression Analysis

The gene expression level was estimated by the RPKM (reads per kilobase million mapped reads) method using HTSeq v0.6.0 (California Institute of Technology, Pasadena, CA, USA) [39]. Pearson’s correlation coefficient between biological replicates was calculated using genes expressed in at least one of the samples. Hierarchical clustering was performed using Pearson’s correlation distance. Analysis of differentially expressed genes was calculated by DESeq (v1.16, Boston, MA, USA) with a false discovery rate (FDR) <0.05 and an absolute value of fold change (FC) >2 [40].

### 4.5. Functional Annotation and Enrichment Analysis

The GO (Gene Ontology, http://geneontology.org/) enrichment of DEGs was implemented using the hypergeometric test, in which the *p*-value was calculated and adjusted as a *q*-value, and data background was genes in the whole genome. GO terms with *q* < 0.05 were considered to be significantly enriched. Pathway analysis of DEGs was performed using the KEGG PATHWAY database (http://www.kegg.jp). The KEGG enrichment of DEGs was also implemented by the hypergeometric test. KEGG terms with *q* < 0.05 were considered to be significantly enriched.

### 4.6. Quantitative Real-Time PCR (qRT-PCR) Analysis

qRT-PCR analysis was performed by using the LightCycler 480 system and the LightCycler 480 SYBR Green Master kit (Roche, Mannheim, Germany) according to the manufacturer’s instructions. The primers sequences used in this study are listed in Table S8. The cycling parameters were as follows: 95 °C for 10 min, 40 cycles of 95 °C for 15 s and 60 °C for 1 min, followed by one cycle of 95 °C for 15 s, 60 °C for 15 s and 95 °C for 15 s. A final step was performed to obtain a melting curve for each PCR product to determine the specificity of amplification. All samples were analyzed in triplicate on the same plate. The expression levels of genes were calculated relative to the expression of the *GAPDH* using the 2^-ΔΔ*C*t^ method.

### 4.7. Analysis of Development-Dependent Gene Expression Patterns

Development-dependent gene expression patterns were analyzed by comparing the union of the DEGs (the RPKMs of the DEGs in all stages were >0) between any two adjacent developmental stages, using the younger developmental stage group as the denominator. Each gene showed at least one significant difference between the developmental stages. By indicating a significant upregulation with “up”, a significant downregulation with “down” and an insignificant difference with “maintain”, there were eight possible patterns, including up-up (UU), up-maintain (UM), up-down (UD), maintain-up (MU), maintain-down (MD), down-up (DU), down-maintain (DM), and down-down (DD).

### 4.8. Statistics

The results are given as the mean ± standard deviation (SD). Statistically significant differences were computed using Student’s *t* test with the statistical software SPSS (Version 20.0, IBM Corp., Armonk, NY, USA). Probability (*p*) values of less than 0.05 were considered to be statistically significant.

## 5. Conclusions

In summary, we revealed a view of gene expression involved in the left-right asymmetric development of the chicken gonads from E6 to D1. A global regression of gene expression profiles was not shown in the development of right gonads. We identified several new candidate genes, GO terms, and pathways that appear to be important for side-specific differential sexual development of gonad in female chicken. Most side-specific genes were located on the autosome rather than the sex chromosome. In addition, this study also revealed eight expression patterns of DEGs during the development of the left and right gonad. Our results will facilitate the study of the molecular characterization of side-specific gonadal development in birds. In the future, the molecular differences of left-right asymmetry between males and females should be characterized.

## Figures and Tables

**Table 1:** Summary of RNA-seq reads mapping to reference genome.

Samples	Clean Reads	Mapped Reads	Mapping Rate (%)	Uniq-Mapped Reads	Uniq-Mapping Rate (%)
E6FR_1	17482191	15924839	91	14374889	82
E6FR_2	17482191	15932034	91	14389796	82
E6FL_1	16816894	15089322	90	13945497	83
E6FL_2	17199818	15466729	90	14299262	83
E12FR_1	16848050	14545265	86	14104709	84
E12FR_2	17179086	14818688	86	14358014	84
E12FL_1	17059514	15157661	89	14570665	85
E12FL_2	16845550	14823214	88	14364057	85
D1FR_1	17983758	15664230	87	15239760	85
D1FR_2	16637905	14418212	87	14093197	85
D1FL_1	16795901	14538377	87	13981293	83
D1FL_2	16717969	14463322	87	14001712	84

Mapped reads: The total number of reads mapped to the reference genome. Mapping Rate: Mapped reads/clean reads. Uniq-mapped: The reads that matched the reference genome in only one position. Uniq-mapping Rate: Uniq-mapped reads/clean reads.

**Table 2:** The most significantly affected gene ontology (GO) terms between right and left ovaries.

Group	GO:Term	Description	Gene_Num De	Gene_Num Back	*p*
E6FR-E6FL		**Biological process**			
	GO:0034587	piRNA metabolic process	7	14	9.4 × 10^−9^
	GO:0043046	DNA methylation involved in gamete generation	6	10	2.7 × 10^−8^
		**Cellular component**			
	GO:0045495	pole plasm	9	12	3.4 × 10^−13^
	GO:0060293	germ plasm	8	11	1.1 × 10^−11^
	GO:0043186	P granule	8	11	1.1 × 10^−11^
E12FR-E12FL		**Biological process**			
	GO:0022402	cell cycle process	293	845	5.5 × 10^−10^
	GO:0007049	cell cycle	381	1159	2.4 × 10^−9^
	GO:0022412	cellular process involved in reproduction in multicellular organism	62	136	2.9 × 10^−7^
		**Cellular component**			
	GO:0005694	chromosome	197	558	1.5 × 10^−8^
	GO:0044427	chromosomal part	170	478	8.3 × 10^−8^
	GO:0000775	chromosome, centromeric region	61	139	7.4 × 10^−7^
		**Molecular function**			
	GO:0016491	oxidoreductase activity	204	587	2.3 × 10^−7^
	GO:0004386	helicase activity	58	130	1.7 × 10^−6^
	GO:0004364	glutathione transferase activity	13	16	4.9 × 10^−6^
D1FR-D1FL		**Biological process**			
	GO:0044699	single-organism process	1861	9535	2.2 × 10^−16^
	GO:0007275	multicellular organismal development	753	3374	2.4 × 10^−13^
	GO:0032501	multicellular organismal process	962	4477	2.9 × 10^−13^
		**Cellular component**			
	GO:0071944	cell periphery	742	3418	1.6 × 10^−10^
	GO:0031224	intrinsic component of membrane	876	4140	4.5 × 10^−10^
	GO:0005886	plasma membrane	723	3341	6.3 × 10^−10^
		**Molecular function**			
	GO:0005215	transporter activity	250	1009	1.4 × 10^−9^
	GO:0022857	transmembrane transporter activity	203	800	8.2 × 10^−9^
	GO:0022892	substrate-specific transporter activity	210	853	6.0 × 10^−8^

Gene_num_de: DEGs in the respective GO term. Gene_num_back: All genes in the respective GO term. At most, three gene ontology categories for biological process, cellular component, and molecular function were shown.

## Supplementary Materials

Supplementary materials can be found at www.mdpi.com/1422-0067/18/6/1299/s1.

## Abbreviations

E6	Embryonic day 6
E12	Embryonic day 12
D1	Post-hatching day 1
DEGs	Differentially expressed genes
FC	Fold change
FDR	False discovery rate
GO	Gene ontology
BP	Biological process
CC	Cellular component
MF	Molecular function
KEGG	Kyoto Encyclopedia of Genes and Genomes
RPKM	Reads per kilobase million mapped reads
qRT-PCR	Quantitative real-time RT-PCR
SD	Standard deviation
UU	Up-up
UM	Up-maintain
UD	Up-down
MU	Maintain-up
MD	Maintain-down
DU	Down-up
DM	Down-maintain
DD	Down-down
