# Supplementary material for: Gene Expression Profiling Reveals Potential Players of Left-Right Asymmetry in Female Chicken Gonads
Source: Int J Mol Sci. 2017 Jun 20;18(6):1299. doi: 10.3390/ijms18061299 (PMC5486120; doi:10.3390/ijms18061299)
Supplement: Supplementary file 1 [file ijms-18-01299-s001.zip › Table S8.pdf]

Table S9. Primers used for qRT-PCR in this study.

Gene	Accession no.	Sequence
GHRH-LR	ENSGALG00000005242	F: AAGGCTGCAGTGGCATTCTT
		R: CAAAGGTCAGCAAAAGCAGTGT
GCGR	ENSGALG00000011219	F: TGCGCTACGTCAAGCTCTTC
		R: TCCGCCTGCACCTCCTT
GABRA3	ENSGALG00000007269	F: CATGTGTCAGACGTCGTCGAT
		R: CAGGCCTTAGGCGGTTATCA
GABRA1	ENSGALG00000001698	F: CAGCTTTGGGCCTGTTTCAG
		R: CATGGGTCCTTTGAATTTTAATCTC
FMO3	ENSGALG00000003316	F: ACGCACGGCTCCATAAGTACA
		R: GGGCGCTTCCTTATCTTGGT
FBP1	ENSGALG00000012613	F: TTCCATTGGGACCATATTTGG
		R: ACCCGCTGCCACAAGATTAC
FAH	ENSGALG00000006482	F: ACTTCTACTCGTCGCGACAACA
		R: TACCCAACAGGCAGATGCAA
ESD	ENSGALG00000016991	F: CAATTTTCCAACCAACCCTGAA
		R: GCCGATACAGATTTGTACTTTCCA
EDNRA	ENSGALG00000010013	F: GGAAGCCTTGTATTTGCGAGTT
		R: TCCCACATCACTCCAATTGGT
CPS1	ENSGALG00000002944	F: ACCATATCACATAGGCAGCAGTGT
		R: GCAATTCACCACGACCGTTT
CALCRL	ENSGALG00000002632	F: CACAGCTTCAGCCACTCAGATG
		R: CGCTGGTGCAGTCGTGATT
ALDOB	ENSGALG00000015544	F: GGGATCTTAGCCGCAGATGA
		R: TTTCTCGAAAAGCACGACGAT
FMO4	ENSGALG00000021066	F: CAGCCGTTTTTGAGGATGGA
		R: CAAATCGACTCTTCAAGGAAGGA
PSPH	ENSGALG00000002397	F: TGCGAAGTTCTGCGGAGTT
		R: TCCTAGTCGTGCCGTTAAAGC
DAM	ENSGALG00000003022	F: GTGGCCAAGTGCTCTGACTTC
		R: ACACCAACACGGCATCAAAG
AGXT	ENSGALG00000020943	F: CTGCCTGCTGCTTGTGGAT
		R: GACTTTCTGGGACCCCGTGTA
PHGDH	ENSGALG00000002988	F: CTCCTCATCTATAGAGCCAAAGCAT
		R: GAGCTGTGGTAGGACTGGAGTTG
PFKL	ENSGALG00000006543	F: TCAGCCTGTCAGGGAACCA
		R: TCATCGAACCTCTTCTCATCCA
HTR1B	ENSGALG00000015895	F: TCCTCGTCTCCATCCTTGTCA
		R: TCCGAGGACAGCCAGATGTC
HHRL	ENSGALG00000004428	F: CCTCTGTCTTCAACATCGTCCTT
		R: ATTGCATTTCTGGTCTTCCCTTT
GLP2R	ENSGALG00000027187	F: TGGAAAATGGAAGTGCAGGAA
		R: TCTGAACTGTCACGCCAAATG

CVH	ENSGALG00000014713	F: AGGCGTGGATGGCTAACTCT
		R: CCAGAACTCCTCCCTCTACCA
PITX2	ENSGALG00000026881	F: CAGAGCATGTTCTCCCCTCC
		R: TAAACGTATGGAGGCGTCGG
TDRD5	ENSGALG00000004138	F: GTTTGCCTCCAAAGGGCAAG
		R: TGTGTGTACTGGAACGTCCG
SLC1A3	ENSGALG00000003582	F: ATGGGAGTCCGAGCAGTAGT
		R: TGGATGTGTCATTGGACGGG
PIWIL1	ENSGALG00000002645	F: GAAGAGCTAGAGCCAGAGCG
		R: TTGCCGCTGTTCAGATGGAT
PITX3	ENSGALG00000005613	F: TCCTACAACAACTGGGCCAC
		R: GTTGAGGCTCGGGCTATTCA
GDF8	ENSGALG00000023961	F: CATGAACCCAGGCACTGGTA
		R: AAATCTCTGCGGGACCGTTT
G0S2	ENSGALG00000023933	F: CCAAAGAGATGCTCAGCCAGA
		R: CAAATCCTCCCGCTTCCGAG
