# Supplementary figures and images for: Gene Expression Profiling Reveals Potential Players of Left-Right Asymmetry in Female Chicken Gonads
Source: Int J Mol Sci. 2017 Jun 20;18(6):1299. doi: 10.3390/ijms18061299 (PMC5486120; doi:10.3390/ijms18061299)

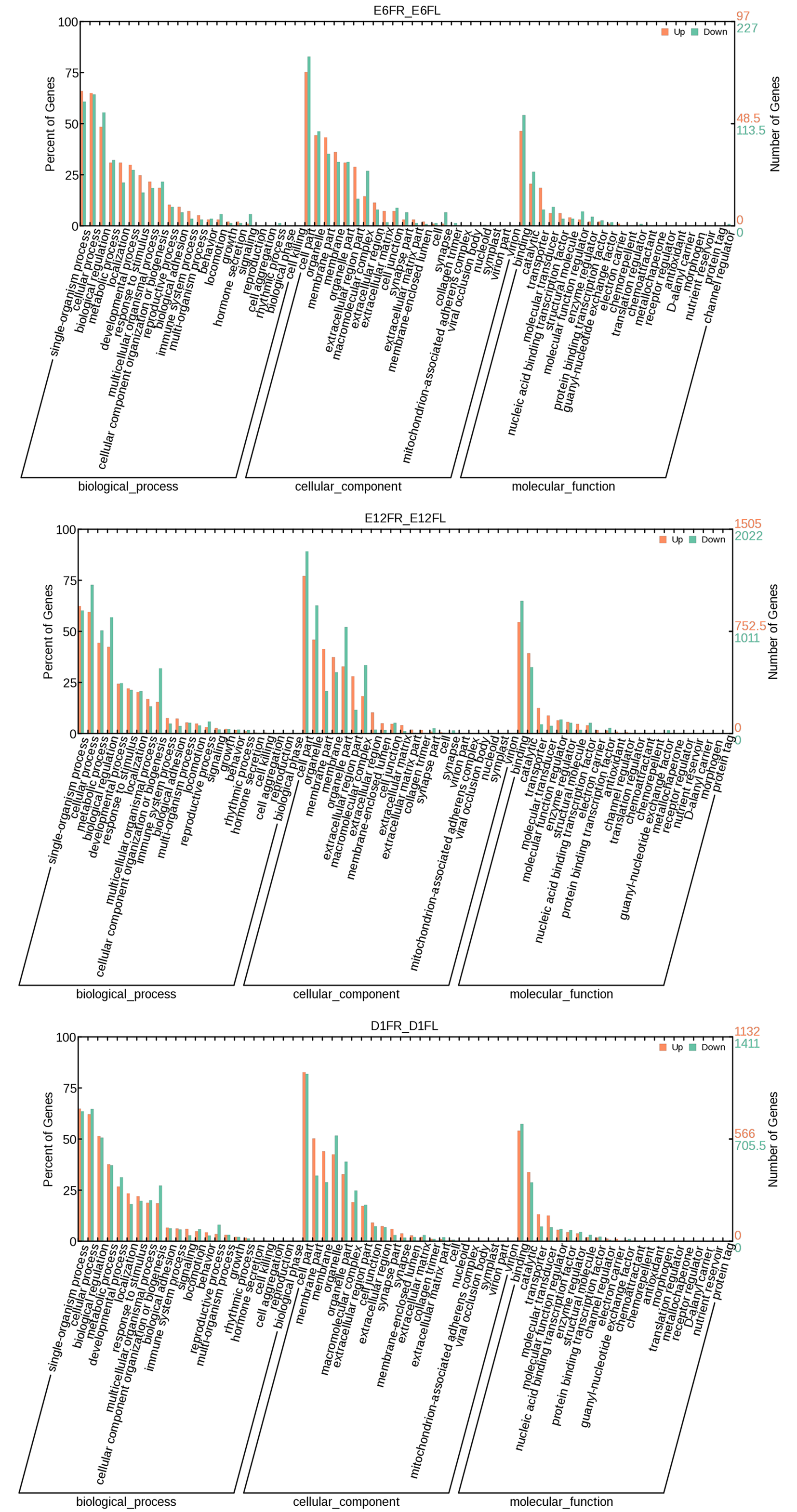

Supplement: Supplementary file 1 [file ijms-18-01299-s001.zip › Figure S1.tif]
